# Practices and promises of Facebook for science outreach: Becoming a “Nerd of Trust”

**DOI:** 10.1371/journal.pbio.2002020

**Published:** 2017-06-27

**Authors:** Craig R. McClain

**Affiliations:** Louisiana Universities Marine Consortium, Chauvin, Louisiana, United States of America

## Abstract

Arguably, the dissemination of science communication has recently entered a new age in which science must compete for public attention with fake news, alternate facts, and pseudoscience. This clash is particularly evident on social media. Facebook has taken a prime role in disseminating fake news, alternate facts, and pseudoscience, but is often ignored in the context of science outreach, especially among individual scientists. Based on new survey data, scientists appear in large Facebook networks but seldom post information about general science, their own scientific research, or culturally controversial topics in science. The typical individual scientist’s audience is large and personally connected, potentially leading to both a broad and deep engagement in science. Moreover, this media values individual expertise, allowing scientists to serve as a “Nerd of Trust” for their online friend and family networks. Science outreach via social media demands a renewed interest, and Facebook may be an overlooked high-return, low-risk science outreach tool in which scientists can play a valuable role to combat disinformation.

## Science outreach via social media

Over the last year, a new political and cultural climate arose in which the prevalence and dissemination of “fake news,” “alternate facts,” and “pseudoscience” rose considerably. The proliferation of “fake news”—the fabrication of sensationalized stories that imitate the style and appearance of real news articles and are published on sites that mimic legitimate outlets [1]—is likely a reflection of the fact that an increasing proportion of the public get their news through social media. Nearly 62% of adults in the United States in 2016 received news from social media, up from 49% in 2012 [2]. The necessity for scientists to engage with the public online is perhaps greater than ever, and over the last decade, scientists have seen increasing calls, from both within and outside the field, to engage with the public [3–5], especially through social media [6–9]. The ease, time, and financial costs of starting a social media account, combined with the potential for very large audiences, makes social media outreach seem very promising. But it’s a big leap from creating a social media account to building a high-profile social media presence buoyed by original content and an engaged audience, which often requires a long-term commitment [9].

Scientists name the considerable time investment as the number one obstacle to participating in public outreach. In a 2008 survey of 325 scientists, “lack of time” was the unanimous factor hindering their participation in public outreach [10]. The same barriers still apply to social media outreach, even though it initially promised a fast and easy way to reach the public. A major problem is that both faculty and administrators consider outreach a volunteer activity without academic reward or incentive for participation. Other priorities such as teaching, research, grant writing, are given precedence. In a more recent survey of 97 scientists [11], respondents felt the pressure of the academy on research productivity left little time for public engagement. Despite the relative ease of using social media platforms, scientists cited the same concern about time restraints as a reason they did not use social media [12]. It seems clear that any strategy to incorporate more scientists into outreach, including social media, must address the problem of the clock.

## The potential and reach of Facebook

The role of Facebook as a source of news and information has become increasingly important as the rise of “fake news” has made clear. Over 66% of Facebook users receive news shared through the site—higher than any other platform except for Reddit [2]. Despite the expanding influence of Facebook, it has received far less attention as a tool for science outreach than Twitter and blogging [8, 9, 13–23]. Indeed, few surveyed scientists believe Facebook is an effective form of online science communication [24]. A sentiment echoed in a study on an institutional Facebook page that concluded “Facebook pages do not offer appropriate social context for learning.” [25]

The peril of ignoring this platform becomes evident when you consider how many people use Facebook—1,790,000,000 monthly users as of the third quarter of 2016, with 81.7% of daily active users residing outside of the US and Canada [26]. Scientists would do well to consider not just how many people are likely to encounter the torrent of baseless scientific information that’s being spread on the site but also what they can do to counter it. And that’s where the payoff for using Facebook as a tool for science outreach comes in. The key to Facebook is the networks that individuals form on the platform: adult users, on average, connect with 338 friends through Facebook.

Daily engagement with Facebook by users appears common, though users primarily consume rather than actively participate in discourse. The average user spends 21 minutes and 6% of their digital time on Facebook [27]. In the US, the daily time spent on Facebook increases to 40 minutes [27] with women visiting the site more often and younger users spending more time on the site [28]. Scientists should note that while Facebook usage is high in both total numbers and frequency of usage, many users may only passively consume rather than actively participate in discourse. Only 44% of users per day liked content posted by their friends, only 31% commented on these posts, and only 10% post status updates to Facebook on a daily basis [29]. However, most Facebook users are actively engaging with their networks on a daily basis: 65% of Facebook users frequently or sometimes share, post, or comment on Facebook. This active engagement is greater than other social media platforms such as Instagram and Twitter [29]. Still, the numbers of likes, shares, and comments may not be the most effective metrics to gauge impact of science-related posts because the passive consumption and exposure to new topics can shift behaviors and perspectives [30].

Key to success is understanding that the reasons people use Facebook vary particularly by gender. A factor analysis identified seven unique uses for Facebook: social connection, shared identities, content, social investigation, social network surfing, and status updating [28]. While the number of male and female users of Facebook are relatively equal [27], usage differs with gender. As a whole, men and women interact with Facebook to view photos and videos from friends (47%), share information with many people at once (46%), read updates from others (39%), and see humorous content (39%) [29]. However, women are more likely to use Facebook to view photos or videos, see entertaining or funny posts, and share information with a large audience [29].

## The Facebook network of scientists

Academics appear to interact with Facebook often—nearly 40% in science and engineering and over 50% in social sciences, arts, and the humanities visit daily [31]. An additional 40–50% are aware of the sites but do not visit regularly [31]. A more recent study finds that 82% of respondents used Facebook [24]. Facebook, in terms of awareness and usage, only falls behind research profiling sites such as Google Scholar, ResearchGate, and Linkedin for scientists and engineers [31]. More recently, however, scientists appear to be more heavily favoring the use of Twitter [24].

In November of 2016, I surveyed scientists via several social media sites examining their usage and behavior on Facebook, including network size and the sharing of science (S1 Text, Supporting Methods). Given the dissemination on social media of the survey through the author’s connections, the respondents are biased toward the fields of biological sciences (21%), marine and aquatic science (22%), environmental and conservation science (11%), and ecology and evolution (31%). Another bias may occur because respondents self-reported posting frequencies and may under- or over-report actual usage. Of the 203 scientists who responded, response rate declined with career stage and gender (and response was greater among females).

The average number of reported friends was 519.33, but the median was lower at 428. Differences in network size were not tied to scientific field, gender, or career stage (S2 Text. Supporting Results). Of these friends, on average 27.5% were reported to be scientists. However, three distinctive clusters of Facebook scientist users were identified: those who connect with nonscientists (most common), mainly other scientists (rare), or a mixture of the two (common). Interestingly, earlier career scientists were much more likely to have Facebook networks that contained nonscientists. Senior scientists were more likely to include scientists as Facebook friends, potentially reflecting a shift in the view of Facebook as a professional networking tool. However, as scientists rise in their scientific careers, their connections with other scientists increase and deepen because of either exposure to new scientific networks or potential isolation within the “ivory tower.” Interestingly, the networks of scientists who self-identified as not having a “traditionally-defined” career within academia (i.e., not labeled as a level of a professor or equivalent) also included more nonscientific members. Scientists’ responses to the survey on Twitter suggest a professional verses personal division on Facebook is fundamental in terms in connections as well as posting habits.

“I keep my personal Facebook separate from work life. Same for Twitter. So no lab news on my Facebook account, no (little) personal stuff [on Twitter].”

“I use Facebook almost exclusively as a friends and family network (Twitter exclusively science)”

Although prior work has shown that many scientists (88%) indicated that they “regularly use Facebook for personal communication where science is shared with interested friends and family,” the survey findings here suggest scientists posting frequencies may be low. The mean number of posts to Facebook reported by survey participants was 16 per month, though most researchers reported well below 6 posts per month. The mean percentage of science posts to Facebook reported by survey participants was 23.6% of posts per month. Many survey respondents posted about science much less than this, with 75% of respondents posting about science less than 33% of total their total monthly Facebook posts. Comments by scientists on social media supported the notion that many scientists have turned to Twitter instead of Facebook for science outreach.

“Posting science to Facebook had become obsolete because of Twitter”

“#Twitter is my primary social media for #science”

“I used to post more science related posts on Facebook before joining Twitter”

**Fig 1:**
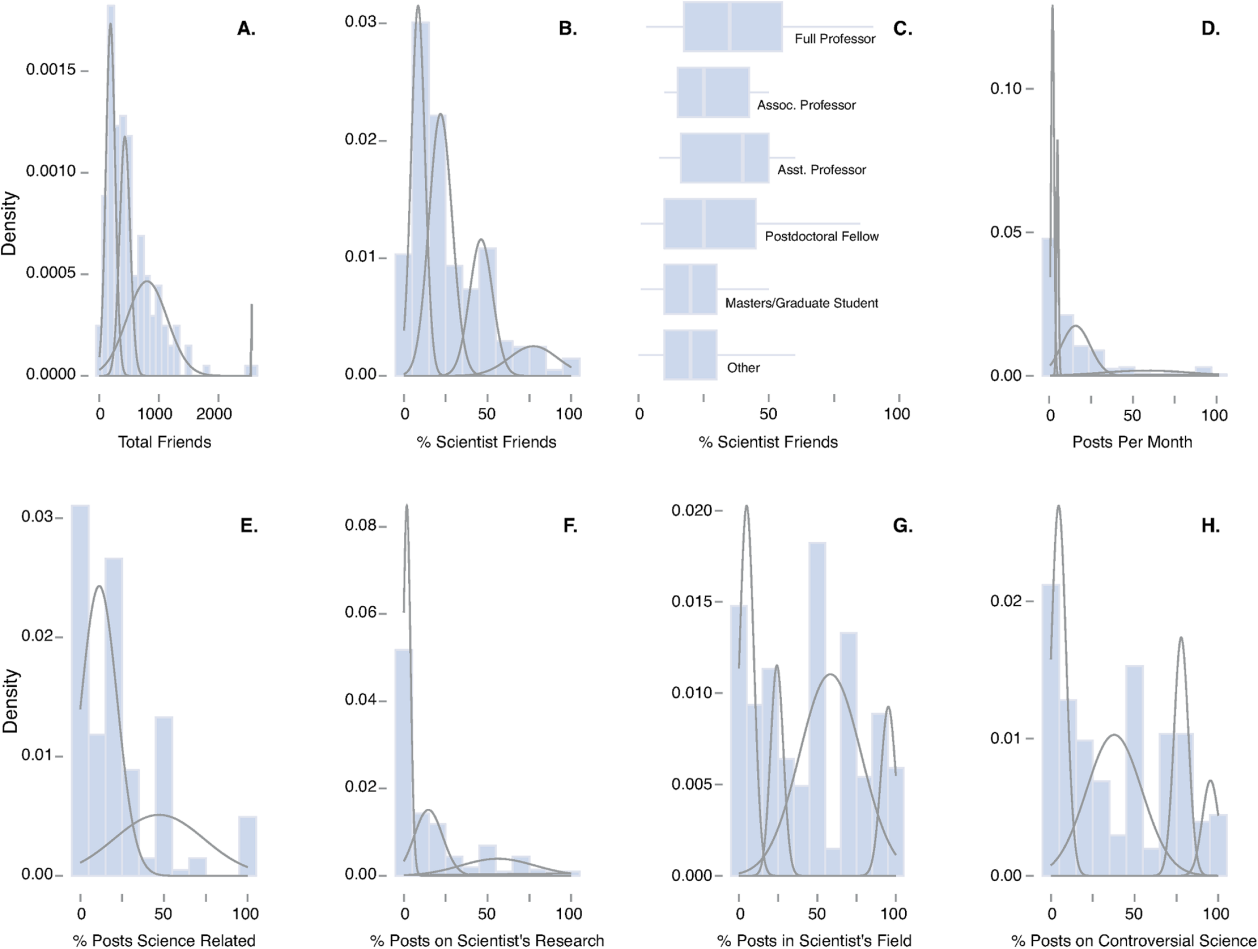
Lines represent normal distributions that best described the data as determined by the Expectation–Maximization Algorithm for Mixtures of Univariate Normals (see S1–S8 Tables). (A) Distribution among scientists of total Facebook friends in their individual networks. (B) Distribution among scientists of percentage of Facebook friends that are scientists. (C) Boxplot of percentage of friends that are scientists in the Facebook networks of survey participants by career stage. (D) Distribution among scientists of total posts per month on Facebook. (E) Distribution among scientists of the percentage of total posts per month that were science related. (F) Distribution among scientists of the percentage of total science posts per month that were related to the personal research of the scientist. (G) Distribution among scientists of the percentage of total science posts per month that were related to the scientific field of the scientist. (H) Distribution among scientists of the percentage of total science posts per month that were related to the culturally controversial science topics.

The scientists surveyed here infrequently post about science (mean = 23.6%). This finding is similar to the finding of another survey reporting that only 25% of scientists posted frequently (52% posted occasionally) about science [24]. Many of those posts were reported not to address the scientist’s own research programs (mean = 17.7% of all science related Facebook posts, Fig 1F). Survey participants had varying practices on sharing science related to their expertise and field. On average, 45.6% of all science-related posts on Facebook were related to the discipline of the researcher. Survey participants also varied in their propensity for sharing culturally controversial science topics (Fig 1H). On average, 40.1% of all science-related posts on Facebook by participants were on controversial topics (e.g., climate change, vaccines, evolution, genetically modified organisms). Survey participants displayed departing practices on addressing controversial science topics with Facebook networks, i.e., with participant groups posting infrequently, moderately frequently, and frequently. Another recent survey found that many scientists are hesitant to engage other Facebook users to correct misrepresentations of science, with only 18% frequently and 40% occasionally posting corrections [24].

## Becoming a Nerd of trust

This study adds to prior work suggesting that scientists appear to be heavily represented on Facebook and many use it regularly to connect with people outside of science [24, 32]. Prior research suggests many scientists already believe in the utility of Facebook to share science with personally connected and interested colleagues, family, and friends [24]. But despite this belief and overall usage of Facebook, the survey results here suggest scientists are still missing a rich opportunity to discuss science with the nonscientists in their networks by actually posting and engaging with their networks. The sample sizes here are limited, but if these results here are representative of the larger population of scientists on Facebook, many scientists may not be taking advantage of the platform as an outreach tool. Here, I argue that Facebook represents an unparalleled and overlooked opportunity.

Models of science outreach on Facebook do exist. For example, several Facebook groups focus on specific patient populations. In particular, these Facebook groups have made it easier for researchers and medical doctors to find patients with rare diseases [33]. However, one study noted in the case of diabetes support groups that while clinically inaccurate recommendations were rare, 27% of posts featured advertising for non-FDA approved, “natural” products, further highlighting the importance and need of scientific experts on Facebook [34]. Established organizations also attract a substantial Facebook following. For example, Scientific American currently has 2,851,129 followers and the National Institute of Health has 312,875. Even independent groups such as my own Deep-Sea News and Dr. Andrew Thaler’s Southern Fried Science receive 15,549 and 8,254 respectively. Indeed, 33% of scientist responding to a survey indicated they administered a Facebook group [24]. The exposure and coordination of social movements including the recent March for Science also testify to the power of Facebook. However, I advocate here that these efforts while successful, at least as measured by followers, are not the same as individual scientists engaging with their personal networks on Facebook, and great opportunity exists in the latter.

### Your personal Facebook audience is large and listening

A large audience already exists on Facebook and the personal networks of individual scientists can be quite substantial. In the survey administered for this study, individual networks ranged for most scientists from 223.5 to 706.0. This is an audience that will likely far exceed any fledgling blog or Twitter account in the first years.

Not only is this audience large but is personally connected to you. The personal relationships that people have on Facebook often transcend the online world, i.e., the depth of connection is greater than other social media outlets. The promise of Facebook is that many scientists are already a “Nerd of Trust” within their network of family and friends [35]. These connections can often traverse ideological affiliations with nearly 20% of liberals maintaining Facebook friendships with conservatives and vice versa [36]. The composition of these social networks is the most important factor that determines the content encountered by social media users [36].

On average the people in an individual’s personal Facebook network, because of familiarity, trust and value their judgment, especially in their specific field [37, 38]. Moreover, Facebook is the new public forum where individuals ask each other for input, e.g., asking a mechanic friend for automotive help, a workout partner for fitness tips, an audiophile for best songs in a genre. This back and forth engagement is an ideal vehicle for online science outreach because it occurs in a community with respect for individual expertise. As this relationship grows, I have personally experienced friends commenting on or asking specific scientific questions on current topics as well as posting current scientific research on my page. It is the latter part that represents one of the most exciting aspects—nonscientists engaging and sharing science.

While posting frequency is a delicate balance between service and annoyance, this reflects the general nature of Facebook; individuals posting about their individual interests. Facebook streams are filled with mentions of politics, fitness, recipes, internet memes, quotes, and humorous videos. People primarily interact with Facebook to connect with the lives of others and for content [28, 29, 39]. A scientist’s life includes science, and posting about the process of that as both a passion and vocation is reasonable. Facebook users predominantly claim their identities implicitly rather than explicitly, i.e., “show rather than tell” [40], a medium thus well suited for science outreach.

### Sharing information is easy and important

Ultimately, scientists need to engage with the social media venues they are already using and enthusiastic about. Facebook is the low-hanging fruit—many scientists already have accounts and are active on a daily to weekly basis. Likewise, it’s easy to post updates, links, photos and videos, especially compared with blogging, and to save time by automating cross-posts from a personal blog or Twitter account, e.g., a service like dlvr.it.

In social media, the role of scientists to make others aware of information and filter this information could potentially be as valuable as generating new content, i.e., a blog post. In the era where fake news and alternate facts are now common, scientists have the expertise and skill set—and some might say, the responsibility [41]—to efficiently and effectively vet online content for scientific accuracy. Any scientist can quickly post a comment or share a link to correct misinformation in the news or on a conversation thread with minimal effort. Scientists should be cautious and respectful in how they respond to misinformation as some strategies may actually reinforce preconceived ideas [42]. However, providing alternative narratives and repeated messages can reduce, but not eliminate, the impact of misinformation [42].

## Facebook for science outreach: The way forward

Realizing the promise of using Facebook for science outreach may require overcoming cultural and technical barriers. Funders may not consider sharing science on a personal Facebook account as a legitimate form of science outreach. Public outreach is part of the broader impacts statement required for a National Science Foundation grant. But reviewers are likely to prefer that research-related content be shared via a blog rather than a personal Facebook account, even though the audience on Facebook is likely to be far greater than the traffic of most fledgling blogs. Another potential issue—and one that could help resolve funders’ hesitation to value social media outreach—is the difficulty of evaluating engagement. Metrics for Facebook are needed that quantify the quality and quantity of engagement with scientific content posted to Facebook. Currently accessing Facebook data is difficult and can often incur a fee. To convince scientists and their funders that it’s worth the effort to counter the proliferation of pseudoscience where it’s most widely disseminated, we need both serious conversations about the legitimacy of personal Facebook accounts for science outreach and the metrics to gauge their success.

## Supporting information

S1 Table Analysis of variance of effects on scientific field, gender, and career stage on total number of Facebook friends. (DOCX)

S2 Table Analysis of variance of effects on scientific field, gender, and career stage on total number of Facebook friends who are scientists. (DOCX)

S3 Table Analysis of variance of effects on scientific field, gender, and career stage on total number of total posts per month on Facebook. (DOCX)

S4 Table Analysis of variance of effects on scientific field, gender, and career stage on percentage of science posts per month on Facebook. (DOCX)

S5 Table Analysis of variance of effects on scientific field, gender, and career stage on percentage of science posts related to scientist’s personal research per month on Facebook. (DOCX)

S6 Table Analysis of variance of effects on scientific field, gender, and career stage on percentage of science posts within scientist’s field or discipline. (DOCX)

S7 Table Analysis of variance of effects on scientific field, gender, and career stage on percentage of science posts related to controversial science topics. (DOCX)

S8 Table Log-likelihoods and means of models of mixtures of normal distributions with different components. (DOCX)

S1 Text Supporting methods for survey on Facebook usage among scientists. (DOCX)

S2 Text Supporting results for survey on Facebook usage among scientists. (DOCX)
