# Supplementary material for: Practices and promises of Facebook for science outreach: Becoming a “Nerd of Trust”
Source: PLoS Biol. 2017 Jun 27;15(6):e2002020. doi: 10.1371/journal.pbio.2002020 (PMC5486963; doi:10.1371/journal.pbio.2002020)
Supplement: S1 Table — (DOCX) [file pbio.2002020.s001.docx]

**S1 Table: Supporting Results**

Analysis of Variance. Effect of scientific field, gender, and career stage on total number of Facebook friends.

Analysis of Variance Table

Response: total_friends

Df Sum Sq Mean Sq F value Pr(>F)

field 12 1232430 102702 0.6123 0.8302

gender 2 124125 62063 0.3700 0.6913

career.stage 5 583115 116623 0.6953 0.6277

Residuals 183 30696274 167739

Residual standard error: 409.6 on 183 degrees of freedom

Multiple R-squared: 0.05943, Adjusted R-squared: -0.03822

F-statistic: 0.6086 on 19 and 183 DF, p-value: 0.897
