# Supplementary material for: Practices and promises of Facebook for science outreach: Becoming a “Nerd of Trust”
Source: PLoS Biol. 2017 Jun 27;15(6):e2002020. doi: 10.1371/journal.pbio.2002020 (PMC5486963; doi:10.1371/journal.pbio.2002020)
Supplement: S2 Table — (DOCX) [file pbio.2002020.s002.docx]

**S2 Table: Supporting Results**

Analysis of Variance. Effect of scientific field, gender, and career stage on total number of Facebook friends who are scientists.

Analysis of Variance Table

Response: percent_scientists

Df Sum Sq Mean Sq F value Pr(>F)

field 12 5677 473.09 1.0250 0.42771

gender 2 176 87.94 0.1905 0.82668

career.stage 5 6412 1282.39 2.7784 0.01913 *

Residuals 183 84464 461.55

Residual standard error: 21.48 on 183 degrees of freedom

Multiple R-squared: 0.1268, Adjusted R-squared: 0.03614

F-statistic: 1.399 on 19 and 183 DF, p-value: 0.1318
