# Supplementary material for: Practices and promises of Facebook for science outreach: Becoming a “Nerd of Trust”
Source: PLoS Biol. 2017 Jun 27;15(6):e2002020. doi: 10.1371/journal.pbio.2002020 (PMC5486963; doi:10.1371/journal.pbio.2002020)
Supplement: S3 Table — (DOCX) [file pbio.2002020.s003.docx]

**S3 Table: Supporting Results**

Analysis of Variance. Effect of scientific field, gender, and career stage on total number of total posts per month on Facebook.

Analysis of Variance Table

Response: post_per_month

Df Sum Sq Mean Sq F value Pr(>F)

field 12 5693 474.40 0.9164 0.5317

gender 2 1889 944.73 1.8250 0.1641

career.stage 5 3321 664.25 1.2832 0.2730

Residuals 183 94733 517.67

Residual standard error: 22.75 on 183 degrees of freedom

Multiple R-squared: 0.1032, Adjusted R-squared: 0.01011

F-statistic: 1.109 on 19 and 183 DF, p-value: 0.3454
