# Supplementary material for: Practices and promises of Facebook for science outreach: Becoming a “Nerd of Trust”
Source: PLoS Biol. 2017 Jun 27;15(6):e2002020. doi: 10.1371/journal.pbio.2002020 (PMC5486963; doi:10.1371/journal.pbio.2002020)
Supplement: S4 Table — (DOCX) [file pbio.2002020.s004.docx]

**S4 Table: Supporting Results**

Analysis of Variance. Effect of scientific field, gender, and career stage on percentage of science posts per month on Facebook.

Analysis of Variance Table

Response: percent_sci

Df Sum Sq Mean Sq F value Pr(>F)

field 12 0.9305 0.077543 1.3125 0.21440

gender 2 0.4010 0.200495 3.3936 0.03571 *

career.stage 5 0.2896 0.057925 0.9805 0.43105

Residuals 183 10.8116 0.059080

Residual standard error: 0.2431 on 183 degrees of freedom

Multiple R-squared: 0.1304, Adjusted R-squared: 0.04011

F-statistic: 1.444 on 19 and 183 DF, p-value: 0.1111
