# Supplementary material for: Practices and promises of Facebook for science outreach: Becoming a “Nerd of Trust”
Source: PLoS Biol. 2017 Jun 27;15(6):e2002020. doi: 10.1371/journal.pbio.2002020 (PMC5486963; doi:10.1371/journal.pbio.2002020)
Supplement: S5 Table — (DOCX) [file pbio.2002020.s005.docx]

**S5 Table: Supporting Results**

Analysis of Variance. Effect of scientific field, gender, and career stage on percentage of science posts related to scientist’s personal research per month on Facebook.

Analysis of Variance Table

Response: percent_per_sci

Df Sum Sq Mean Sq F value Pr(>F)

field 12 3606 300.54 0.5307 0.89288

gender 2 1327 663.26 1.1713 0.31228

career.stage 5 7112 1422.47 2.5120 0.03156 *

Residuals 183 103627 566.27

Residual standard error: 23.8 on 183 degrees of freedom

Multiple R-squared: 0.1041, Adjusted R-squared: 0.01112

F-statistic: 1.12 on 19 and 183 DF, p-value: 0.3345

Tukey HSD

diff lwr upr p adj

Full Professor-Associate Professor 1.010043 -24.532341 26.55243 0.9999973

Other-Associate Professor 3.800731 -15.809993 23.41146 0.9935033

Full Professor or Equivalent-Associate Professor 9.181516 -14.779363 33.14240 0.8793801

Masters or Graduate Student-Associate Professor 12.302557 -5.686246 30.29136 0.3637772

Assistant Professor-Associate Professor 16.599106 -2.515063 35.71328 0.1291340

Other-Full Professor 2.790688 -19.904315 25.48569 0.9992585

Full Professor or Equivalent-Full Professor 8.171473 -18.372951 34.71590 0.9493210

Masters or Graduate Student-Full Professor 11.292513 -10.016632 32.60166 0.6477899

Assistant Professor-Full Professor 15.589063 -6.678271 37.85640 0.3370934

Full Professor or Equivalent-Other 5.380785 -15.518343 26.27991 0.9764323

Masters or Graduate Student-Other 8.501825 -5.145403 22.14905 0.4720150

Assistant Professor-Other 12.798375 -2.301337 27.89809 0.1477595

Masters or Graduate Student-Full Professor or Equivalent 3.121040 -16.264266 22.50635 0.9972873

Assistant Professor-Full Professor or Equivalent 7.417590 -13.016317 27.85150 0.9017914

Assistant Professor-Masters or Graduate Student 4.296550 -8.626988 17.22009 0.9305620
