# Supplementary material for: Practices and promises of Facebook for science outreach: Becoming a “Nerd of Trust”
Source: PLoS Biol. 2017 Jun 27;15(6):e2002020. doi: 10.1371/journal.pbio.2002020 (PMC5486963; doi:10.1371/journal.pbio.2002020)
Supplement: S7 Table — (DOCX) [file pbio.2002020.s007.docx]

**S7 Table: Supporting Results**

Analysis of Variance. Effect of scientific field, gender, and career stage on percentage of science posts related to controversial science topics.

Analysis of Variance Table

Response: percent_controversial

Df Sum Sq Mean Sq F value Pr(>F)

field 12 7945 662.10 0.6110 0.8312

gender 2 2085 1042.51 0.9621 0.3840

career.stage 5 1719 343.72 0.3172 0.9022

Residuals 183 198292 1083.56

Residual standard error: 32.92 on 183 degrees of freedom

Multiple R-squared: 0.05594, Adjusted R-squared: -0.04208

F-statistic: 0.5707 on 19 and 183 DF, p-value: 0.9233
