# Supplementary material for: Practices and promises of Facebook for science outreach: Becoming a “Nerd of Trust”
Source: PLoS Biol. 2017 Jun 27;15(6):e2002020. doi: 10.1371/journal.pbio.2002020 (PMC5486963; doi:10.1371/journal.pbio.2002020)
Supplement: S8 Table — (DOCX) [file pbio.2002020.s008.docx]

**S8 Table: Supporting Results**

Log-likelihoods of models of mixtures of normal distributions with different components (k=2-4, models did not converge at k>4) as implemented by the EM Algorithm for Mixtures of Univariate Normals (R-package with the mixtools package). NA indicates a lack of convergence at that level of k. Means for the normal distributions are provided for the best-fit model (in bold) as determined by log-likelihoods.

| **Model** | **Total Facebook Friends** | **Percent of Friends That Are Scientists** | **Total Posts Per Month** | **Percent of Posts on Science** | **Percent of Posts on Scientist's Research** | **Percent of Posts in Scientist's Field** | **Percent of Posts on Controversial Science** |
| --- | --- | --- | --- | --- | --- | --- | --- |
| 1 | -1556.08 | -913.94 | -922.88 | -939.42 | -932.10 | -990.09 | -992.64 |
| 2 | -1465.68 | -872.77 | -800.20 | **-902.26** | -812.31 | -963.74 | -946.45 |
| 3 | -1456.24 | -866.75 | -765.29 | NA | **-793.85** | -948.55 | -932.36 |
| 4 | **-1441.39** | **-860.00** | **-755.12** | NA | NA | **-946.80** | **-923.53** |
|  |  |  |  |  |  |  |  |
| **Means** |  |  |  |  |  |  |  |
| 1 | 9.3 | 8.5 | 1.6 | 11.3 | 1.8 | 4.5 | 4.6 |
| 2 | 21.9 | 22.0 | 4.8 | 47.1 | 14.7 | 38.5 | 37.8 |
| 3 | 39.3 | 46.2 | 16.6 | - | 56.2 | 75.9 | 77.8 |
| 4 | 83.8 | 77.4 | 62.9 | - | - | 94.6 | 95.3 |
