# Supplementary material for: Practices and promises of Facebook for science outreach: Becoming a “Nerd of Trust”
Source: PLoS Biol. 2017 Jun 27;15(6):e2002020. doi: 10.1371/journal.pbio.2002020 (PMC5486963; doi:10.1371/journal.pbio.2002020)
Supplement: S1 Text — (DOCX) [file pbio.2002020.s009.docx]

**S1 Text. Supporting Methods**

In November of 2016, a survey was administered via several social media sites including the author’s personal accounts, those of the popular marine blog [www.deepseanews.com](http://www.deepseanews.com), ecological and evolutionary listservs, and through several popular hashtags on Twitter, e.g. #scicomm. The purpose was to quantify the network sizes and usage of Facebook for publishing science content. The questions asked included:

1. What is your career stage?
2. What is your field of study?
3. What is your gender?
4. How many friends are in your Facebook network?
5. What percentage of friends in your network are scientists? (as defined by you)
6. How many times in the last 30 days did you post to Facebook?
7. How many times in the last 30 days did you make a status update about science?
8. What percentage of the science posts you share to Facebook reference your specific research?
9. What percentage of the science posts you share to Facebook deal with culturally controversial topics in science (e.g. climate change, vaccines, evolution, GMOs)?
10. What percentage of the science posts you share to Facebook deal with culturally controversial topics in science (e.g. climate change, vaccines, evolution, GMOs)?
