# Supplementary material for: Practices and promises of Facebook for science outreach: Becoming a “Nerd of Trust”
Source: PLoS Biol. 2017 Jun 27;15(6):e2002020. doi: 10.1371/journal.pbio.2002020 (PMC5486963; doi:10.1371/journal.pbio.2002020)
Supplement: S2 Text — (DOCX) [file pbio.2002020.s010.docx]

**S2 Text. Supporting Results**

Two hundred and three scientists responded. Response rate generally declined with career stage (Master or Graduate Student n=75, Postdoctoral Fellow n=45, Assistant Professor or Equivalent n=18, Full Professor or Equivalent n=15, Associate Professor or Equivalent N=12, Other N=38). Responses from females were also greater than males (Female N=127, Male N=73, Prefer Not to Say N=3).

All answers and numbers were self-reported by participants, e.g. the percentage of friends in the network that are scientists is a count provided by the survey participant and not a direct measure by the researcher.

*Facebook Network Size*

The average number of friends was 519.33 (range: 5-2563) but the median was lower (428) and the distribution was significantly right-skewed (Skew=1.89, D'Agostino skewness test: p<<0.0001, Figure 1A). Using the EM Algorithm for Mixtures of Univariate Normals (implemented in R-package with the mixtools package), log-likelihoods were compared of models of mixtures of normal distributions with different components (k=2-4, models did not converge at k>4). The best model fit four components (Table S8) indicating some group differences in the effort and or ability for scientists in forming Faceboook networks. However, these differences appeared not to be tied to scientific field, gender, or career stage (Table S1; ANOVA: p-value=0.897).

*Scientific verses Public Network Size*

Of these friends, on average 27.5% (range 0-100%) were scientists. However, the distribution is noticeably non-unimodal (Figure 1B). A comparison of models of mixtures of normal distributions with different components found the best model fit four components (Table S8). This suggests much different perceptions and behaviors among the surveyed scientists in Facebook usage ranging from groups using Facebook predominately for connecting with non-scientists (most common, mean=8.5% & 22.0% of friends who were scientists) to other scientists (rare, mean=77.4%) connections to a mixture of the two (common, mean=46.2%).

Career stage impacted these relationships (Table S2; ANOVA: p-value=0.0191, Figure 1C) but gender and discipline did not (p-value=0.4277 & 0.8267 respectively).

*General Activity on Facebook*

The mean number of posts to Facebook by survey participants was 16 per month. However, the distribution was heavily right-skewed (Skew=2.34, D'Agostino skewness test: p<<0.0001, Figure 1D). A comparison of models of mixtures of normal distributions indicated potentially four populations/groups within the distribution based on posting frequency. However, most researchers fell well below 6 posts per month. The total number of Facebook posts per month by scientists was not dependent on gender, career stage, or field (Table S3).

*Science Posts on Facebook*

The view that many scientists do not use Facebook either for the dissemination of science or building science networks is supported by the survey data on science posting as well. The mean percentage of science posts to Facebook by survey participants was 23.6% of posts per month (Figure 1E) with 25% of participants posting science less than 4% and 75% posting science less than 33% of total Facebook posts per month. The distribution was right-skewed (Skew=1.46, D'Agostino skewness test: p<<0.0001). However, the distribution was bimodal (Table S8) indicating a group that posts rarely about science (~11% of posts) and those who post often (~50%). Career stage and field did not impact these finding but gender had a marginally significant impact (Table S4). This significant impact of gender is driven primarily by an increase in science posting frequency on Facebook of participants who preferred not to identify a gender.

*Facebook Posts on Scientist’s Own Research and Field*

Most survey participants also rarely shared information about their personal research on Facebook (Figure 1F) with the mean percentage of posts per month being 17.7% of science-related Facebook posts. However, the median was much less (median=5%) and the distribution was right skewed (Skew=1.65, D'Agostino skewness test: p<<0.0001). ) The comparison of models of mixtures of normal distributions indicated potentially three populations/groups within the distribution, two comprising the majority of survey participants of which posted infrequently about personal research and a rare group of participants that posted more frequently (Table S8). An ANOVA (Table S5) indicated a significant effect for career stage but Tukey HSD post-hoc test did not reveal significant differences between individual categories.

Survey participants had varying practices on sharing science related to their expertise and field on Facebook. On average, 45.6% of all science related posts on Facebook were related to the discipline of the researcher (Figure 1E). The distribution is visually multimodal. Log-likelihoods were compared of models of mixtures of normal distributions with different components. The best model fit four components suggesting different attitudes and practices on type of science shared by researchers on Facebook (Table S8). An ANOVA (Table S6) indicated a significant effect for career stage but Tukey HSD post-hoc test did not reveal significant differences between individual categories.

*Posting on Culturally Controversial Topics in Science*

Survey participants also had varying practices on sharing culturally controversial science topics on Facebook (Figure 1H). On average, 40.1% of all science related posts on Facebook by participants were on controversial topics. Like distribution posts that were in the participant’s field, the distribution for the posts pertaining to controversial issues is also visually multimodal. Log-likelihoods were compared of models of mixtures of normal distributions with different components revealing four distributions (Table S8). This implies departing practices on addressing controversial science topics with Facebook networks. An ANOVA (Table S7) indicated no significant effect for career stage, gender, or field.
